# C. A. Kingsbury, M.D., D.D.S.

**Published:** 1891-11

**Authors:** 


### Obituary.

### C. A. KINGSBURY, M.D., D.D.S.

Dr. Kingsbury, of Philadelphia, died, after a short illness, October 3, 1891.

Dr. Kingsbury was born in East Windsor, Conn., in 1820. Being left an orphan at an early age, he removed to Northern New Hampshire, where he attended the local schools until he was sixteen, when he became a teacher, at the same time pursuing his studies at the Wesleyan Academy, Massachusetts, and Newbury, Vt. He began the study of dentistry at Trenton, N. J. He removed to Philadelphia in 1839, and entered actively in the practice of his profession.

His interest in everything that tended to advance dentistry in a truly scientific direction was deep and lasting, and when the Philadelphia Dental College was founded, in 1863, Dr. Kingsbury was elected to fill the chair of Dental Histology and Operative Dentistry. Having served in this capacity for some time, he resigned, and was elected Emeritus Professor in the same school.

Dr. Kingsbury had an extensive knowledge of men in many stations of life. His activity seemed to have no limit, and probably this hastened his death, as he worked continuously over the chair during June of this year, being more pressed with practice than at any period of his life.

His many-sided character led him away from the narrow paths of recreation ordinarily trodden by professional men. He had an intense love of nature, and usually spent his summer vacation in remote places salmon-fishing and hunting. It was while on a trip West he was taken sick, and which proved to be the close of his life-work. His love of angling was only excelled by his prototype Isaak Walton, and his greatest pleasure was found in places and streams not frequented by the idle summer crowd. This taste led him to an intimate knowledge of the habits of the denizens of the air and water. He took great interest in the efforts to stock our rivers with fish, and was a member of the Pennsylvania Fish and Game Protective Association.

Interest in religious matters was a marked trait in his character, but he was broad enough not to confine this to his own persuasion.

He was pre-eminently a social man, and continued to the hour of his death a member of the Philadelphia Dental Club, where his extended information and varied experience added much to the intellectual enjoyment of those reunions.

In his professional work he stood in the front rank. Associated as he had been with the most prominent men in dentistry, contemporary with Townsend, Harris, Arthur, and other leaders, he had advanced with its growth, ever conservative, but still ready to adopt new methods when proved valuable.

He was a member of several dental societies, and was one of the original members of the Odontological Society of Pennsylvania, and continued active in its work to the close.

He took a positive interest in the "men before the mast," and was president of the Seamen's Aid Society.

To the writer and many others his death comes as a personal loss. The number of those who have been called from active professional life has been in recent months, as a writer expresses it, "truly appalling;" and it does certainly appear as though death had never before been so active in our ranks.

Dr. Kingsbury took part at the "Patriarchs' Dinner" in New York, and made an address upon that occasion. This reunion, so happily planned by Dr. Kingsley and others, was to him a satisfactory meeting, bringing him near those who had worked and labored for the elevation of his chosen calling.

The dental profession will join in the feeling that it has parted with an earnest and consistent worker, and an exemplar to those younger men who must not only fill his place, but others daily becoming vacant in the profession.

He leaves a wife, one daughter, and two sons to mourn his loss.

The funeral services took place at the Methodist church, Broad and Arch Streets, Philadelphia. A large number of the dental fraternity were present. The interment was at Allentown, N. J.

